# The oncogenic role of JC virus T antigen in lens tumors without cell specificity of alternative splicing of its intron

**DOI:** 10.18632/oncotarget.3507

**Published:** 2015-03-10

**Authors:** Wen-feng Gou, Shuang Zhao, Dao-fu Shen, Xue-feng Yang, Yun-peng Liu, Hong-zhi Sun, Jun-sheng Luo, Hua-chuan Zheng

**Affiliations:** ^1^ Cancer Research Center, Key Laboratory of Brain and Spinal Cord Injury of Liaoning Province, and Laboratory Animal Center, The First Affiliated Hospital of Liaoning Medical University, Jinzhou, China; ^2^ Department of Oncological Medicine, The First Affiliated Hospital of China Medical University, Shenyang, China

**Keywords:** JC virus, T antigen, oncogenesis, transgenic mouse, lens tumor

## Abstract

JC virus (JCV), a ubiquitous polyoma virus that commonly infects the human, is identified as the etiologic agent for progressive multifocal leukoencephalopathy and some malignancies. To clarify the oncogenic role of JCV T antigen, we established two transgenic mice of T antigen using either α-crystallin A (αAT) or cytokeratin 19(KT) promoter. Lens tumors were found in high-copy αAT mice with the immunopositivity of T antigen, p53, β-catenin and N-cadherin. Enlarged eyeballs were observed and tumor invaded into the brain by magnetic resonance imaging and hematoxylin-and-eosin staining. The overall survival time of homozygous mice was shorter than that of hemizygous mice (p<0.01), the latter than wild-type mice (p<0.01). The spontaneous salivary tumor and hepatocellular carcinoma were seen in αAT5 transgenic mice with no positivity of T antigen. KT7 mice suffered from lung tumor although JCV T antigen was strongly expressed in gastric epithelial cells. The alternative splicing of T antigen intron was detectable in the lens tumor of αAT mice, gastric mucosa of KT mice, and various cells transfected with pEGFP-N1-T antigen. It was suggested that JCV T antigen might induce carcinogenesis at a manner of cell specificity, which is not linked to alternative splicing of its intron. Both spontaneous lens and lung tumor models provide good tools to investigate the oncogenic role of JCV T antigen.

## INTRODUCTION

JC virus (JCV) is a human non-enveloped polyomavirus with small, closed, circular, double-stranded DNA in 5130-bp length and encodes 6 genes: 3 viral capsid proteins (VP1, VP2 and VP3), agnoprotein, small t-Ag and large T-Ag [1]. Although JCV replication is restricted to the glial cells and lymphoid cells, which express such JCV transcriptional factors as Jun, NF-1, GF-1, Sp1, Sμbp-2, Purα, and YB-1, the virus enters tonsillar tissue and persists quiescent in the kidney and lymphoid tissue during latency [2]. JCV existence in raw sewage and the abundance in a critical component of the JCV receptor (terminal α 2, 6-linked sialic acid) in normal lung suggest that both ingestion of contaminated water and inhalation of air droplet may represent possible entrances of JCV into the human population [3, 4]. JCV infection initiates binding to the JCV-sensitive cell surface under immunosuppressive conditions. In permissive infection, JCV replicates DNA, results in lytic infection and becomes known for over 40 years as a causative agent for progressive multifocal leukoencephalopathy. However, non-permissive cells don't allow the viral replication, leading to an abortive infection or cell transformation [1, 5].

Current evidence highlights JCV T antigen as the main oncogenic protein. T antigen, a large nuclear multifunctional phosphoprotein for viral DNA replication, binds to and breaks DNA to promote the unwinding of double helix and recruitment of cell proteins because T-antigen can serve as ATPase, helicase, and polymerase and orchestrate the assembly and function of replication protein A and DNA polymerase-α [6]. T antigen can inactivate p53 and the members of pRb family, resulting in deregulation of cell cycle checkpoints and elimination of p53-mediated pro-apoptotic activity [7]. Additionally, T antigen exerts its oncogenic activity by deregulating the Wnt signaling pathway through stabilization of β-catenin and its interaction with the IGF-IR signaling system for cellular transformation [8-10]. It was found that T antigen inhibited AP2 binding to the Bag3 promoter, down-regulating Bag3 expression and subsequently inhibiting apoptosis because Bag3 initiated apoptosis as molecular co-chaperones of Hsc70/Hsp70 [11]. Reportedly, Bag3-induced autophagy was associated with degradation of JCV T antigen [12]. Reviriego-Mendoza et al.[13] demonstrated that T antigen bound to β-transducin-repeat containing protein-1 and 2 (βTrCP1/2) and disrupted further proteasomal degradation of β-catenin. T antigen expression was associated with a metastatic phenotype of colorectal cancer, which might partly be mediated through the AKT/MAPK pathway. Meanwhile, T antigen could induce the structural chromosome aberrations and genomic instability as a mutator [14].

Intravenous or intracranial inoculation of JCV into the experimental animals could cause astrocytomas, glioblastomas, neuroblastomas and medulloblastomas [15]. Transgenic mouse strain expressing JCV *T-antigen* exhibited pituitary adenoma in approximately 50% of animals and some of them developed to malignant peripheral nerve sheath tumors [16, 17]. Although a direct link between JCV infection and human carcinogenesis remains elusive, JCV has been recently suggested to correlate with various types of human cancers, including colorectal, prostatic and esophageal cancers, brain tumors, head neck squamous carcinoma, bronchopulmonary, gastrointestinal, anal, cervical and urothelial cancer [18-28]. These reports may indicate that JCV plays some role in human epithelial tumorigenesis as oncovirus. To further its oncogenic role, we established two transgenic mice expressing JCV T antigen in gastric mucosa or lens epithelium. Subsequently, we for the first time found that the insertion of JCV T antigen into genome might cause carcinogenesis with cell specificity, but alternative splicing of T antigen intron was of no cell specificity.

## RESULTS

### JCV T antigen induced the spontaneous lens tumor of transgenic mice

**Figure 1:**
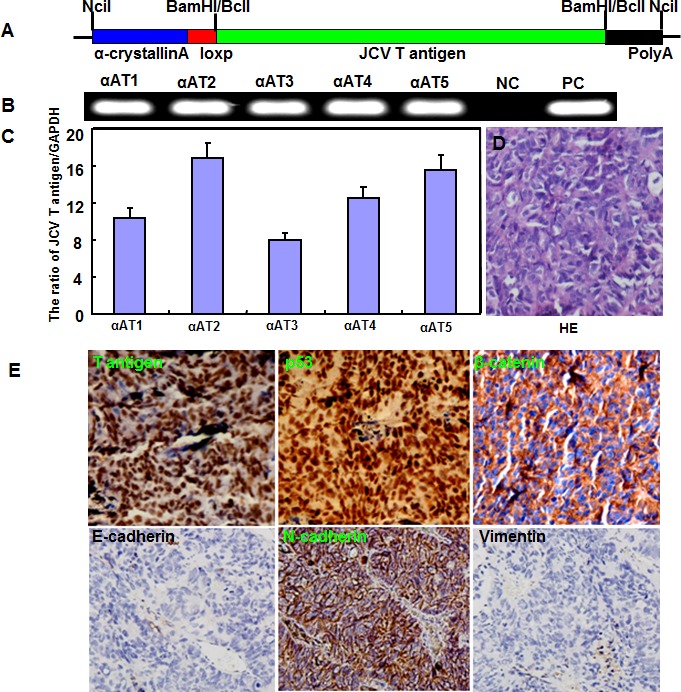
The spontaneous lens tumor of JCV T antigen transgenic mice by α-crystallin A promoter The transgenic mice of JCV T antigen was established according to schematic diagram using α-crystallin A promoter (A). We found five founders with T antigen positive by PCR of mouse tail DNA (B). Among them, αAT2 and αAT5 showed higher copies of JCV T antigen by real-time PCR (C). The mice of both founders showed lens tumor evidenced by hematoxylin and eosin staining (D). Immunohistochemically, there appeared positivity of JCV T antigen (nuclear), p53 protein (nuclear), β-catenin (membrane and cytoplasm) and N-cadherin (membrane and cytoplasm), while negativity of E-cadherin and Vimentin (E).

**Figure 2:**
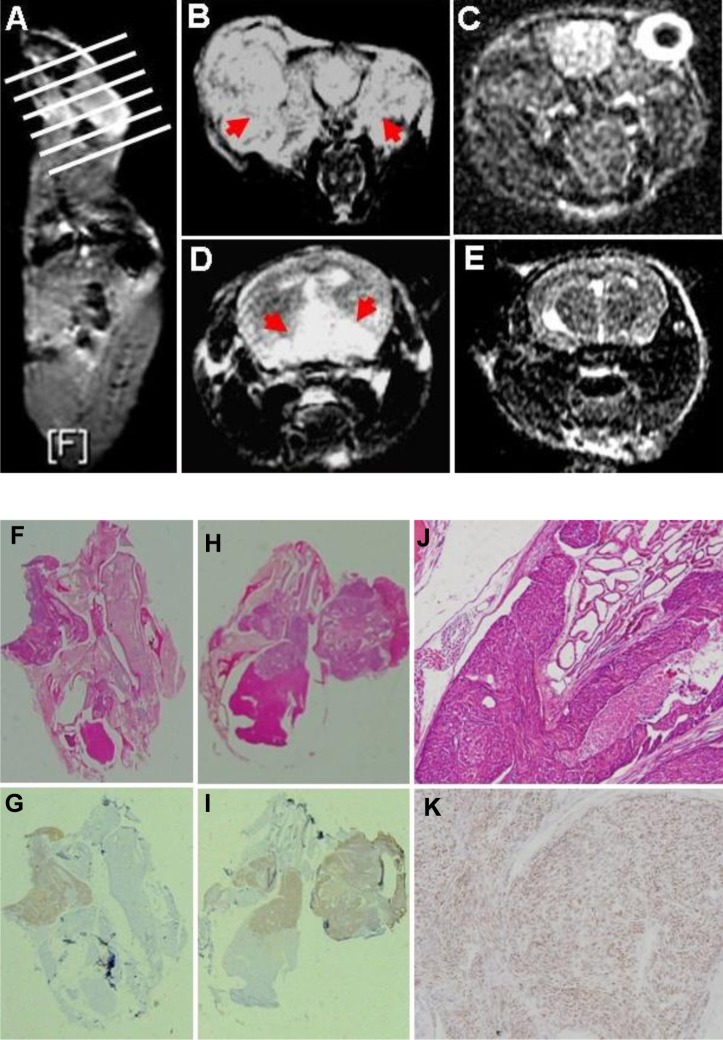
The lens tumor can invade into the brain by magnetic resonance imaging (MRI) and histological examination An αAT2 mouse with lens tumors of both eyes was scanned according the white line orientation by MRI (A). The enlarged eyeballs with tumor (B, axial T2 weighted image, red arrow) were observed in comparison to the wild-type mouse(C, axial FSPGR image). The part tumor invaded into the brain (D, axial T2 weighted image, red arrow), while wild-type mouse showed no any alteration (E, FSPGR image). Histologically, αAT2 mice were subjected to routine HE examination (F, H, J) and the brain invasion was found. Immunohistochemically, the tumor cells showed positive reactivity to JCV T antigen (G, I, K; nuclear).

**Figure 3:**
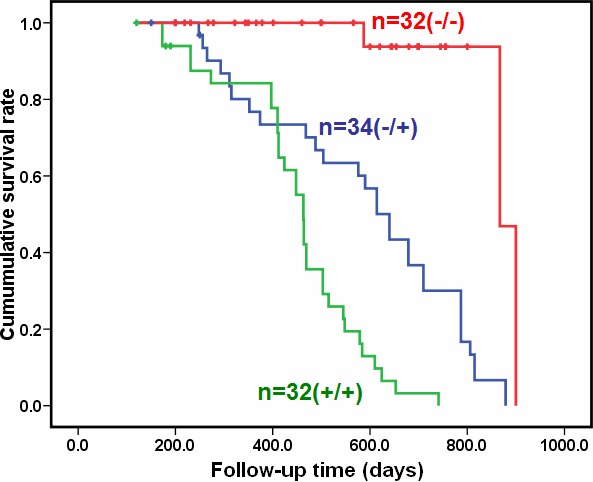
The overall survival comparison for the homozygous and hemizygous αAT2 transgenic mice Kaplan-Meier curves for cumulative survival rate of transgenic mice with lens tumor according to the genotype of JCV T antigen. The overall survival time of homozygous mice was shorter than that of hemizygous mice (*p* < 0.01). The survival rates of both transgenic mice were lower than that of wild-type mice (*p* < 0.01).

The transgenic mice of JCV T antigen were established using α-crystallin A promoter specific for lens epithelial cells (Fig. 1A and 1B). Among them, both αAT2 and αAT5 founders showed higher copies of JCV T antigen by real-time PCR (Fig. 1C) and suffered from lens tumor evidenced by HE staining (Fig. 1D). Immunohistochemically, there appeared immunopositivity of T antigen, p53, β-catenin and N-cadherin, while immunonegativity of E-cadherin and Vimentin in lens tumor cells (Fig. 1E). Magnetic resonance imaging (MRI) showed that the enlarged eyeballs with tumor (Fig. 2A-C) were observed and tumor invaded into the brain (Fig. 2D and 2E), which was also evidenced by HE staining and T antigen immunostaining (Fig. 2F-K). According to Kaplan-Meier curves and log-rank test, the overall survival time of homozygous αAT2 mice was shorter than that of hemizygous mice (*p* < 0.01, Fig. 3). The survival rates of both transgenic mice were lower than that of wild-type mice (*p* < 0.01, Fig. 3).

**Figure 4:**
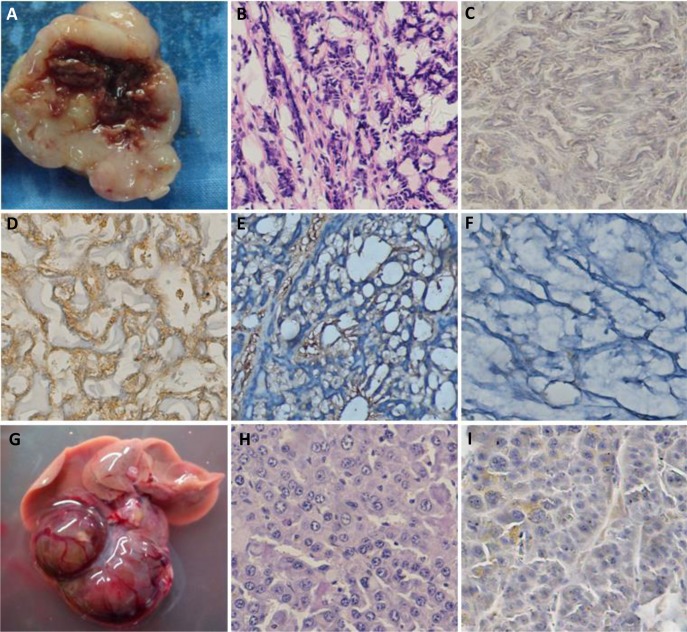
The spontaneous salivary carcinoma and hepatocellular carcinoma in αAT5 JCV T antigen transgenic mice In an αAT5 mouse, there was a head and neck tumor (A and B), which had positive expression of α-SMA (D), negative expression of JCV T antigen (C), cytokeratin 7 (E) and thyroglobulin (F). In another αAT5 mouse, liver tumors (G) were found to be hepatocellular carcinoma (H) with no expression of JCV T antigen (I).

As shown in Fig. 4, both spontaneous head and neck tumor and liver tumor were seen in αAT5 mice. According to HE and immunohistochemistry, it was diagnosed as salivary carcinoma because of positive expression of α-SMA, negative expression of cytokeratin 7 and thyroglobulin. The liver tumor was diagnosed as hepatocellular carcinoma according to the microscopy of HE slide. Interestingly, JCV T antigen was not detected in both tumor cells.

### JCV T antigen overexpression caused spontaneous lung cancer of transgenic mice

**Figure 5:**
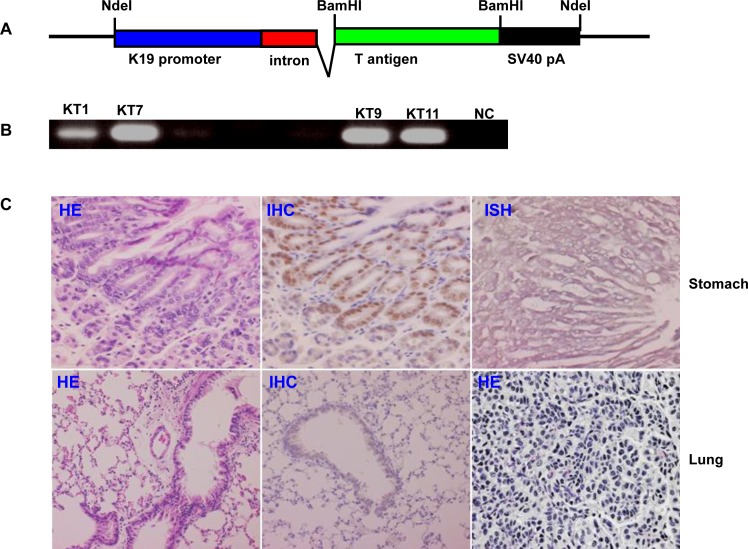
The JCV T antigen transgenic mice by cytokeratin 19 promoter The transgenic mice of JCV T antigen was established according to schematic diagram using cytokeratin 19 promoter (A). The eleven founders were screened for T antigen positive by PCR of mouse tail DNA (B). Both stomach and lung of KT7 mouse were histologically examined by hematoxylin and eosin staining (C). KT7 showed the positivity of T antigen at both protein and mRNA levels in gastric epithelial cells by immunohistochemistry and in situ hybridization respectively (C). Additionally, T antigen was also observed in lung bronchial epithelial cells. A 16-month KT7 mouse suffered from lung cancer (C). NC, negative control; HE, hematoxylin-eosin staining; IHC, immunohistochemistry; ISH, in situ hybridization.

The transgenic mice of JCV T antigen were established using cytokeratin 19 promoter, which is specific for gastric epithelial cells (Fig. 5A and 5B). The mRNA and protein of JCV T antigen were strongly expressed in gastric epithelial cells of KT7 transgenic mouse by immunohistochemistry and in situ hybridization respectively, while no morphological alteration was observed in gastric mucosa (Fig. 5C). Additionally, JCV T antigen was detectable in bronchial epithelium in KT7 founder, which suffered from lung cancer at the age of 16 months (Fig. 5C).

### No cell specificity of alternative splicing of JCV T antigen in eukaryocytes

**Figure 6:**
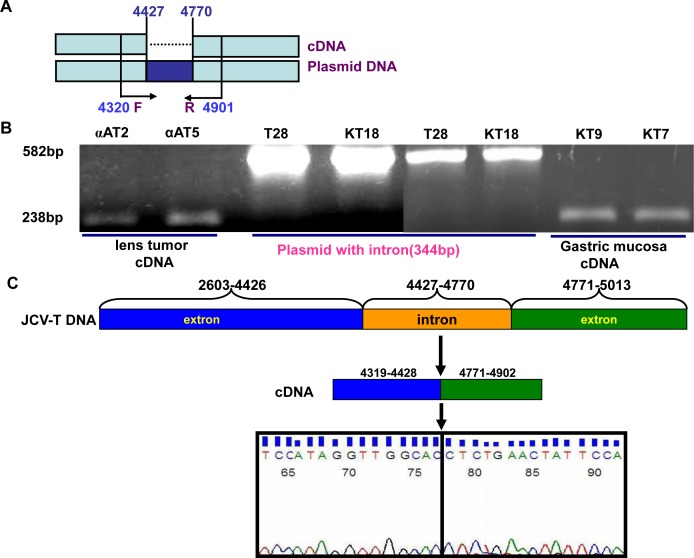

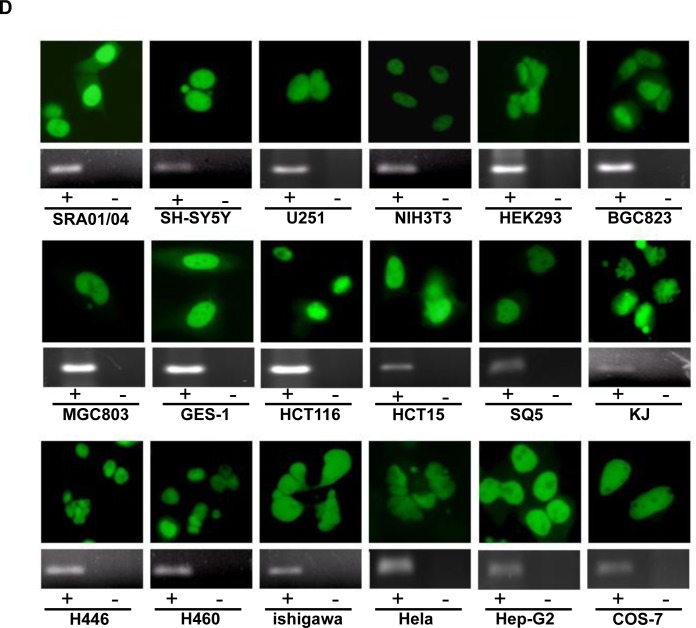
No cell specificity of alternative splicing of JCV T antigen The primers were designed targeting the intron of T antigen (A). The cDNAs from lens tumor of αAT-2, -5 mouse, and gastric mucosa of KT7 or KT9 mouse were amplified and the intron deletion was observed with pbluescipt-T antigen (T28) and K19-T antigen (KT18) plasmids as positive control (B). Direct sequencing showed that the intron deletion of T antigen amplicon occurred (C). Finally, cells were transfected with pEGFP-N1-T antigen and fused GFP fluorescence was observed in the nuclei (D). RT-PCR showed intron deletion in all transfectants (+), with the maternal cell as negative control (−, D).

To verify the alternative splicing, we designed the primers targeting the intron of T antigen (Fig. 6A). Intron deletion was found in the lens tumor cDNAs of αAT-2 and -5 as well as the mucosa cDNA of KT7 and KT9 mice (Fig. 6B). The sequence alignment indicated that the intron deletion occurred (Fig. 6C). The carcinoma or normal cells originating from the ectoderm, mesoderm and endoderm were subjected to the transient transfection of pEGFP-N1-T antigen. GFP fluorescence indicating T antigen expression was observed in the nuclei of all tested cells (Fig. 6D). The alternative splicing of T antigen intron was detectable in all transfectants by RT-PCR (Fig. 6D), followed by direct sequencing (data not shown).

## DISCUSSION

Human cells experience a constant barrage of oncogene activation, thus to create the convertion of a human embryonic cell into a tumor cell, including H-Ras mutation, the ectopic expression of the telomerase catalytic subunit, and SV40 T antigen [5]. In line with our previous report [29], JCV T antigen for the first time induced lens tumor upon its specific expression in lens epithelial cells in two founders of transgenic mice, which showed high copies of JCV T antigen. It was proposed that the insertion of JCV T antigen in genomic DNA might be closely linked to lens tumorigenesis although it is believed to be a highly neurotrophic virus whose genes are preferentially expressed in astrocytes and oligodendrocytes of human brain. Additionally, we prepared a transgenic mouse of JCV T antigen using K19 promoter, specific to gastric epithelium [30], and found T-antigen-positive pulmonary tumors in 16-month-old KT7 mice. In combination of the findings, we hypothesize that JCV T antigen is likely to be important in pushing normal epithelial cells along the multistep path to malignancy. Immunostaining showed positive expression of N-cadherin, but negative expression of E-cadherin and Vimentin, indicating that spontaneous lens tumor might origin from mesenchymal cells or experience epithelial-mesenchymal transition.

JCV T antigen also plays an important role in the stabilization of β-catenin by small GTPase Rac 1 [31], disrupts further proteasomal degradation of β-catenin by binding to βTrCP1/2 [13], and interacts with ß-catenin for the nuclear entry of ß-catenin, thereby up-regulating the nuclear gene expression of c-myc and Cyclin D1 [32]. In addition, JCV T antigen can bind to and inactivate p53 protein with the dysfunction of cell cycle checkpoints [34]. In the present study, β-catenin expression was merely restricted to the membrane and cytoplasm of tumor cells in agreement with our previous report [33]. We speculate that β-catenin is mainly involved in the cell adhesion in JCV T antigen-overexpressing tumor cells of transgenic mice. Although nuclear p53 was strongly expressed in lens tumor cells, we could not differentiate the wild- or mutant-type p53 because JCV T antigen was reported to cause genomic instability, such as EGFR mutation in lung tumor of transgenic mice [33]. High p53 expression also might be attributable to feedback overexpression as a tumor suppressor.

The advantage of the model is only localized to lens so as to grossly observe the tumor alteration. In our animal model, advanced lens tumor appeared to invade into the brain through foramina opticum according to the findings of MRI and HE staining, consistent with a previous study [29]. The survival time of homozygous mice was shorter than that of hemizygous mice, the latter than wild-type ones, which was significantly associated with tumor growth. Therefore, it is possible to employ the spontaneous tumor model into experimental treatment for advanced JCV T antigen-related tumors. Interestingly, there occurred spontanous hepatocellular carcinoma and salivary carcinoma in αAT5 mice, but not in the other founders and wild-type mice. In contrast, JCV T antigen was not detected in both kinds of tumor cells. We suspected that the insertion of JCV T antigen might activate other oncogenes, whose interaction with environmental factors results in carcinogenesis.

In KT mice, T antigen is more expressed in gastric epithelial cell than bronchial epithelial cells, but lung cancer is observed. This demonstrated cell specificity for the JCV-T antigen-induced carcinogenesis excluding the leakage of K19 promoter. In the following experiment, we constructed full-length and GFP-fused T antigen expressing plasmid and transfected it into various cells, including mesoderm-ectoderm- or endoderm-derived normal or cancer cells. The alternative splicing of T antigen intron was found in all cells, suggesting that the intron deletion of JCV T antigen is of no cell specificity during its oncogenesis. The 344bp intron, not triple, will cause shift mutation in T antigen transcript if not deleted. Therefore, it wais for the first time concluded that the cell specificity of T antigen-induced carcinogenesis is not due to alternative splicing of T antigen intron.

In summary, JCV T antigen can induce spontanous lens tumor of transgenic mice, which might be employed to study the molecular mechanisms and clinical therapeutics of JCV T antigen-related cancer. The cell specificity of T antigen-induced tumorigenesis is not correlated with the alternative splicing of T antigen, but correct expression of its encoding protein. The detailed mechanisms of T antigen-related oncogenesis need be further investigated in the future.

## MATERIALS AND METHODS

### Transgenic mice

Transgenic mice were generated by conventional method as described previously [35]. Briefly, pBluescript-T antigen was constructed using pBluescipt vector and pBS-JCVMad1 (kindly provided by Prof. Sawa, Hokkaido University) as reported in our previous work [36]. A 2.4kb bclI/bclI DNA restriction fragment of Kozak-sequence-containing JCV T antigen was inserted after the α-crystallin A promoter (Prof. Nakamura, Kawasaki Medical University) by *BamH*I (Fig. 1A), or into pK19 plasmid (kindly provided by Prof. Oshima, Cancer Institute, Kanazawa University) between cytokeratin 19 promoter (K19) and SV40 polyA (Fig. 5A) by *BamH*I. Either of two expressing plasmids was microinjected into the fertilized eggs of the F1 (C3H and C57BL6) hybrid females crossed with C57BL/6 male.

### Cell culture and plasmid transfection

Human lens epithelial cell (SRA01/04), human neuroblastoma cell (SH-SY5Y), human glioblastoma cell (U251), mouse embryonic fibroblast (NIH3T3), human embryonic kidney cell (HEK293), human gastric carcinoma cells (BGC-823 and MGC-803), human gastric epithelial cell (GES-1), human colorectal carcinoma cells (HCT-15 and HCT-116), human lung carcinoma cells (SQ-5, KJ, H446 and H460), human endometrial adenocarcinoma cell (Ishigawa), human cervical carcinoma cell (Hela), human hepatocellular carcinoma cell (Hep-G_2_) and monkey kidney cell (Cos-7) were maintained in RPMI 1640, MEM, or DMEM medium supplemented with 10% fetal bovine serum, 100 units/ml penicillin, and 100 μg/ml streptomycin, in a humidified atmosphere of 5% CO_2_ at 37°C.

T antigen was amplified using a pair of primers (Forward: 5′-CGACCGCTCGAGATGGAC AAAGTGCTGAATAGG-3′; Reverse: 5′-GACCGG AATTCGTTTTGGGGGAGGGGTCTTTGG-3′) and pBluescript-T antigen as a template. The products were digested and inserted into pEGFP-N1 vector between *Xho*I and *EcoR*I. All the cells were transiently transfected by pEGFP-N1-T antigen and observed under fluorescence microscopy. After that, cells were harvested by centrifugation, rinsed with PBS, and subjected to RNA extraction.

### Polymerase chain reaction (PCR)

DNA was extracted from the mouse tail by proteinase K digestion and phenol/chloroform. Total RNA was extracted from lens tumor, gastric mucosa, and cell lines using QIAGEN RNeasy mini kit (QIAGEN, Hilden, Germany) according to the manufacturer's protocol. Two micrograms of total RNA were subjected to cDNA synthesis using the Avian Myeloblastosis Virus (AMV) transcriptase and random primer (Takara, Otsu, Japan). DNA was amplified by PCR targeting T antigen (Forward: 5′-TGGCCTGTAAAGTTCTAGGCA-3′ and Reverse, 5′-GCAGAGTCAAGGGATTTACCTT C-3′). Real-time PCR was performed using SYBR Premix Ex Taq^TM^ II kit. The mouse GADPH (Forward: 5′-ACATACTCAGCACCGGCCTC-3′ and Reverse: 5′-TATGACTCCACTCACGGCAAA-3′) was employed as an internal control. To confirm the alternative splicing, we designed the primers (Forward: 5′-TCATCATCACTGGCAAAC-3′ and Reverse: 5′-GCAAAGAACTCCACCCT-3′) around the intron of T antigen (Fig. 6A).

### Magnetic resonance imaging (MRI)

Mice were positioned head-up in a 3.0 T MRI scanner (GE sigma 3.0T HDXT, USA) with high Q animal-specific wrist coil used to ensure a high signal to noise ratio and an uniform magnetic field. Mice were sedated with pentobarbital sodium (2%) during MRI procedures. We performed unenhanced T1-weighted (axial 3D-FSPGR; FOV = 6.0; slice thickness 1 mm, TR 320 ms; IR(inverse recovery) prep; TI = 350; TE in phase; FA = 15°; Bandwidth: 31; Slices: 30; Freq: 272, Phase: 256; BW 31.25Hz; NEX = 8) and T2-weighted imaging (TR = 4000; TE = 108/Ef, EC:1/1 31.2KHZ, HEAD/FL:AAC, FOV:6*5/Z; ZIP:512; 1.0thk/0.2sp, 7/08:40, NEX: 416* 288/8; ED/TRF). All images were collected with field of view of 50 mm, slicethickness 1mm with a final in-plane resolution of 100 × 100 μm.

### Histopathology

All tissues were fixed in 4% neutralized formaldehyde, embedded in paraffin and incised into 4 μm sections. The brain was subjected to decalcification using 10% EDTA (pH7.4) between fixation and embedding. These sections were stained by hematoxylin-and-eosin (HE) method to confirm their histological diagnosis and other microscopic characteristics.

### Immunohistochemistry(IHC)

Consecutive sections were deparaffinized with xylene, rehydrated with alcohol, and subjected to antigen retrieval by irradiating in TRS (DAKO) for 5 min with microwave oven (Oriental rotor Lmt. Co.). Five percent bovine serum albumin was then applied for 1 min to prevent non-specific binding. The sections were incubated with anti-T antigen (Calbiochem, USA), anti-p53 (DAKO, USA), anti-β-catenin (BD Transduction, USA), anti-N-Cadherin (Abcam, USA), anti-Vimentin(DAKO, USA), anti-α-SMA (Abcam, USA), anti-cytokeratin 7(DAKO, USA), anti-E-cadherin (Abcam, USA) or anti-thyroglobulin (DAKO, USA) antibody for 20 min, then treated with the Envison-PO (DAKO, USA) antibody for 20 minutes. All the incubation was performed in the microwave oven for intermittent irradiation as described previously [37]. After each treatment, the slides were washed with TBST (10mM Tris-HCl, 150mM NaCl, 0.1% Tween 20) three times for 5 min. All slides were colored with 3, 3‘-diaminobenzidine (DAB) and counterstained with mayer's hematoxylin. Omission of the primary antibody was used as a negative control and appropriate positive controls were utilized as recommended by the manufacturers.

### *In situ* hybridization(ISH)

To perform RNA-DNA ISH for *T antigen*, a digoxygenin-labeled T antigen probe was made using the above-mentioned primers and pBluescript-T antigen by PCR. The sections were deparaffinized and digested with proteinase K at 37°C. Twenty μL probe in hybridization buffer was added to each slide. After coverslipping, heating, and overnight incubation in a humidified chamber at 42°C, sections were rinsed in TBST and incubated with anti-digoxygenin antibody conjugated with alkaline phosphatase (Roche) at 37°C. The slides were then washed and immersed in solution II (100 mM Tris-HCl pH9.5, 100 mM NaCl and 50 mM MgCl_2_) followed by exposure to NBT/BCIP as a chromogen. Finally, counterstaining was performed using methyl green.

### Statistical analysis

Statistical evaluation was performed using Mann-Whitney U to differentiate the means of different groups. Kaplan-Meier survival plots were generated and comparisons between survival curves were made with the log-rank statistics. *P <* 0.05 was considered as statistically significant. SPSS 10.0 software was employed to analyze all data.
